# Draft Genome Sequence of *Enterococcus faecium* Strain 58m, Isolated from Intestinal Tract Content of a Woolly Mammoth, *Mammuthus primigenius*

**DOI:** 10.1128/genomeA.01706-15

**Published:** 2016-02-11

**Authors:** Artemiy Goncharov, Semyon Grigorjev, Alena Karaseva, Viktoria Kolodzhieva, Daniil Azarov, Yana Akhremenko, Lidia Tarasova, Alexei Tikhonov, Alexey Masharskiy, Lyudmila Zueva, Alexander Suvorov

**Affiliations:** a FSBSI Institute of Experimental Medicine, Saint Petersburg, Russian Federation; b North-Western State Medical University named after I. I. Mechnikov, Saint Petersburg, Russian Federation; c Saint Petersburg State University, Saint Petersburg, Russian Federation; d Zoological Institute of the Russian Academy of Sciences, Saint Petersburg, Russian Federation; e M. K. Ammosov North-Eastern Federal University, Yakutsk, Russian Federation

## Abstract

*Enterococcus faecium* 58m is a putative ancient nonpathogenic strain isolated from the intestinal content of an adult woolly mammoth (*Mammuthus primigenius*). Here, we report its draft genome sequence, consisting of 60 contigs. *In silico* genomic analysis was performed to determine the genetic features and pathogenic potential of this microorganism.

## GENOME ANNOUNCEMENT

Enterococci are common commensal members of gut communities in mammals and can also be isolated from a variety of plants, animals, and other environmental sources. However, some strains have recently been recognized as emerging nosocomial pathogens (1). The evolutionary history of enterococci still remains under discussion; therefore, it is important to compare the strains from various natural habitats.

*Enterococcus faecium* strain 58m is a putative ancient bacterial isolate obtained from the digestive tract content of the so-called Malolyakhovskiy mammoth (*Mammuthus primigenius* [Blumenbach, 1799]), whose partial carcass was excavated by staff of the Institute of Applied Ecology of the North, North-Eastern Federal University (Yakutsk, Russian Federation) in May 2013 (2). The remains of this animal were dated by an accelerator mass spectrometry (AMS) method at the Center for Isotope Research of Groningen University at 28,610 ± 110 years of age. A pure culture was obtained by growing the isolates on blood agar plates at 37°C. Bacteria from each individual colony were grown overnight in tryptic soy broth, pelleted by centrifugation at 5,000 × *g* for 10 min, and genomic DNA was extracted using the QIAamp Fast DNA stool minikit (Qiagen). Genomic DNA was used to construct a sequencing library employing a NEBNext Ultra DNA library prep kit (New England BioLabs, Ipswich, MA). Sequencing was performed on an Illumina MiSeq with the 301-cycle MiSeq reagent kit version 2, to achieve 150× average genome coverage. The quality of the raw sequence data was checked using FastQC (http://www.bioinformatics.babraham.ac.uk/projects/fastqc/).

The resulting nucleotide sequences were assembled *de novo* into 60 contigs using the *Platanus* 1.2.1 software (3). Only 37 contigs were >1,000 bp in size. The *N*_50_ contig length was 243,081 bp, the largest contig assembled was 443,734 bp, and the shortest contig was 213 bp. The draft genome sequence consists of 2,754,403 bp, with a mean G+C content of 38.0%.

Genomic analysis was done using the RAST annotation server (4), Blast algorithms, ARAGORN (5), and BAGEL3 (6). The results obtained with RAST showed that there are 341 subsystems denoted in the chromosome, which represent only 48% of the assigned sequences. A total of 2,679 coding sequences (CDSs) and 80 structural RNAs (63 tRNAs) were predicted. None of the known virulence genes were identified. BAGEL3 software analysis demonstrated the presence of two bacteriocin genes, including acidocin LF221B (contig 59) and enterolysin A (contig 48) in this strain, demonstrating the biotechnological potential of this microorganism.

Interestingly, a set of genes related to uptake mechanism for nickel and cobalt, which are present in all environmental isolates but rarely observed in enteric isolates, was localized in contig 46. However, the genes for xyloside and lactose utilization, which are common among enteric genomes but absent in environmental genomes and IS*16* (a marker of nosocomial strains) (7, 8), were also found.

The obtained data may be useful for future comparative genomic studies on the evolution of host adaptation of *E. faecium*.

### Nucleotide sequence accession numbers.

This whole-genome shotgun project has been deposited at DDBJ/EMBL/GenBank under the accession no. LGAN00000000. The version described in this paper is version LGAN00000000.1.
